# Reliability and methodology of quantitative assessment of harvested and unharvested patellar tendons of ACL injured athletes using ultrasound tissue characterization

**DOI:** 10.1186/s13102-019-0124-x

**Published:** 2019-07-19

**Authors:** Carla S. Pereira, Rafael C. G. Santos, Rod Whiteley, Taija Finni

**Affiliations:** 1 ASPETAR Orthopaedic and Sports Medicine Hospital, Sports City Street, Inside Aspire Zone, Al Buwairda St, Doha, PO Box 29222, Qatar; 2 Faculty of Sport and Health Sciences, Biology of Physical Activity, Neuromuscular Research Center, University of Jyväskylä, Jyväskylä, Finland

**Keywords:** Anterior cruciate ligament, Echo-types distribution, Graft, Tendon quality, UTC

## Abstract

**Background:**

Ultrasound tissue characterization (UTC) imaging has been previously used to describe the characteristics of patellar and Achilles tendons. UTC imaging compares and correlates successive ultrasonographic transverse tendon images to calculate the distribution of four color-coded echo-types that represent different tendon tissue types. However, UTC has not been used to describe the characteristics of patellar tendons after anterior cruciate ligament reconstruction (ACLR). The aim of this cross-sectional study was to assess the intra and inter-rater reliability of the UTC in unharvested and harvested patellar tendons of patients undergoing ACLR.

**Methods:**

Intra and inter-rater reliability of both UTC data collection and analysis were assessed. Ten harvested and twenty unharvested patellar tendons from eighteen participants were scanned twice by the same examiner. Eleven harvested and ten unharvested patellar tendons from sixteen participants were scanned and analyzed twice by two different examiners. Twenty harvested and nineteen unharvested patellar tendons from twenty-three participants were analyzed twice by two examiners.

**Results:**

Quantification of the proportion of echo-types I, II, III and IV in the areas of interest: (1) patella apex, (2) proximal tendon, (3) mid tendon, (4) distal tendon, and overall tendon of harvested and unharvested patellar tendons all displayed excellent intra-rater reliability (ICC_2,1_: 0.94 to 0.99), excellent inter-rater reliability for harvested and unharvested patellar tendon scanning and analysis (ICC_2,1_: 0.89 to 0.98), and excellent inter-rater reliability for analysis (ICC_2,1_: 0.95 to 0.99). Intra-rater reliability for the measure of volume was good (ICC_2,1_: 0.69 harvested, 0.67 unharvested), whilst mixed results were observed for the measure of mid tendon thickness (ICC_2,1_: 0.88 harvested, 0.57 unharvested). Inter-rater reliability for scanning and analysis was good for volume (ICC_2,1_: 0.67) and excellent for thickness (ICC_2,1_: 0.97), while the inter-rater reliability for analysis was fair to poor for volume (ICC_2,1_: 0.59 harvested, 0.30 unharvested), and excellent to poor for mid tendon thickness (ICC_2,1_: 0.85 harvested, 0.24 unharvested).

**Conclusion:**

UTC imaging is a reliable tool to characterize the quality of most aspects of unharvested and harvested patellar tendons in subjects undergoing ACLR.

## Background

Ultrasound tissue characterization (UTC) has been used to assess the integrity of tendon structure in animals and humans. [1–8] UTC captures contiguous transverse ultrasound images over the length of the tendon and semi- quantifies the stability of the echotexture over successive transverse ultrasonographic images. [1–3] Four different echo-types have been proposed to discriminate the underlying tendon tissue types; type I = intact and aligned collagen bundles; type II = discontinuous, swollen and wavy collagen bundles; type III = loose matrix; and type IV = amorphous matrix. [2] The validation of this method to date has originally been based on histopathologic studies of the superficial digital flexor tendons of horses, [1, 2, 9], and subsequently the use of UTC has expanded to human tendons. [3, 5, 6] Reliability of UTC imaging in both healthy and pathological tendons has demonstrated high intra- and inter-observer reproducibility for both acquisition and analysis. [3, 10]

Studies using UTC imaging have documented alterations in tendon appearance in the presence of clinically diagnosed Achilles tendinopathy, [3, 7, 11–18] patellar tendinopathy, [11, 18] systemic disease such as diabetes, [19] after platelet-rich plasma (PRP) injection, [4] and after different therapeutic exercise programs. [5, 6, 12, 14]

Anterior cruciate ligament (ACL) injuries are one of the most devastating injuries encountered in sports medicine due to the likely requirement of surgery, and the extended recovery and rehabilitation period following the injury. Where surgical reconstruction of the injured ligament is decided (ACL reconstruction - ACLR), surgeons may choose from a range of possible grafts to repair the torn ligament, including allograft – from cadavers or synthetic, and autograft – when either a portion of the quadriceps tendon, hamstrings tendons (Hst), or frequently, the patellar tendon (BTB) is harvested. [20] BTB autograft has garnered increased attention and popularity in recent decades, which has been attributed to the hypothesis that BTB grafts provide superior post-operative stability via its bone-to-bone attachments [21, 22]. However, BTB grafts have been associated with increased donor site morbidity, particularly anterior knee pain and quadriceps weakness have been reported [23–25]. Potentially increased understanding of the effects of BTB grafts on tendon structure may help negate the potential side effects of this surgical approach. While UTC has documented reliability and normative data for typical anterior knee pain populations (echo-type I (%) 58 ± 7; echo-type II (%) 34 ± 5; echo-types III (%) 6 ± 4; echo-type IV (%) 3 ± 2) [10], there are no normative or reliability data for those undergoing ACLR using a BTB graft. To establish the utility of interventions for these populations and to understand meaningful changes of the tendon tissue characteristics as they relate to symptoms, reliability and normative data need to be documented in this population. Therefore, the aim of the current study was to assess the intra- and inter-rater reliability of UTC imaging in harvested patellar tendons after ACLR and to provide normative values for this population.

## Methods

### Participants

The patellar tendons assessed in this study were from participants who sought conservative or surgical treatment for an ACL injury at Aspetar, Orthopaedic and Sports Medicine Hospital, Doha, Qatar. Thirty-seven male athletes registered within Qatar’s sporting federations regularly attending Aspetar Orthopaedic and Sports Medicine Hospital for rehabilitation following ACL injury and/or ACLR during the period of February to August 2018 were invited to participate in the study. Patients were deemed suitable to participate in the study if they were: male, had a diagnosed ACL tear confirmed by magnetic resonance imaging or a previously performed ACLR, and agreed to take part in one or more phases of this study and to be assessed by different examiners and/or on different days (Table 1).

**Table 1:** Participants’ graft type, sport, patellar tendon investigated and participation time in the different analysis

Participant’s number	Graft	Sports	Intra-rater Harvested	Intra-rater Unharvested		Inter-rater Acquisition & Analysis		Inter-rater Harvested	Inter-rater Unharvested	
			Involved	Involved	Uninvolved	Involved	Uninvolved	Involved	Involved	Uninvolved
1	Hst	Football		10.5 M					10.5 M	
2	BTB Revision	Football				3 M		3 M		
3	BTB	Table tennis				6 W		6 W		preop
4	BTB	Futsal							preop	preop
5	BTB	Football	1Y					1Y		
6	BTB	Football				preop	preop			
7	Hst	Volleyball				preop				
8	BTB	Football				6 M		6 W, 6 M	preop	
9	BTB	Football					6 M			
10	Allograft	Basketball				6 M				
11	BTB	Handball						6 W, 6 M	preop	
12	BTB Revision	Football	6 W					6 W		
13	BTB Revision	Football			3 M, 4.5 M					4.5 M
14	Conservative	Football				preop				
15	BTB	Handball				6 M				
16	Hst	Football		6 M	6 M				6 M	6 M
17	BTB	Football	9 M		9 M			9 M		9 M
18	BTB	Football							preop	preop
19	BTB	Football	9 M		10.5 M			9 M		10.5 M
20	BTB	Football	7.5 M					7.5 M		
21	Hst	Handball		4.5 M	4.5 M				4.5 M	4.5 M
22	Hst	Football		6 M	6 M					6 M
23	BTB	Hockey				4.5 M	4.5 M			
24	BTB	Football	4.5 M					4.5 M		
25	BTB	Football	3 M		3 M			3 M		3 M
26	BTB	Football				6 W	6 W			
27	Hst	Cycling		6 W	6 W					
28	BTB	Handball				6 M	6 M	6 W, 6 M	preop	
29	BTB	Basketball	6 W		6 W			6 W		6 W
30	BTB	Football	6 W		6 W			6 W		
31	BTB	Rugby					preop			
32	Hst	Handball		3 M						
33	BTB	Sky diving				6 W				
34	BTB	Football				6 W	6 W	6 W		
35	BTB	Football	3 M		3 M			3 M		
36	Conservative	Football		preop	preop					
37	BTB	Football				3 M		3 M		

“preop”: pre-operation. “W”, “M”, and “Y” denote weeks, months, and years post-operative respectively

*BTB* Bone patellar tendon bone graft, *Hst* Hamstring graft

Written informed consent was obtained from each participant or legal guardian. Ethical approval was obtained by ethical committee of the Anti-Doping Laboratory Qatar Research Office (2017000227).

### Ultrasound tissue characterization (UTC)

**Fig. 1:**
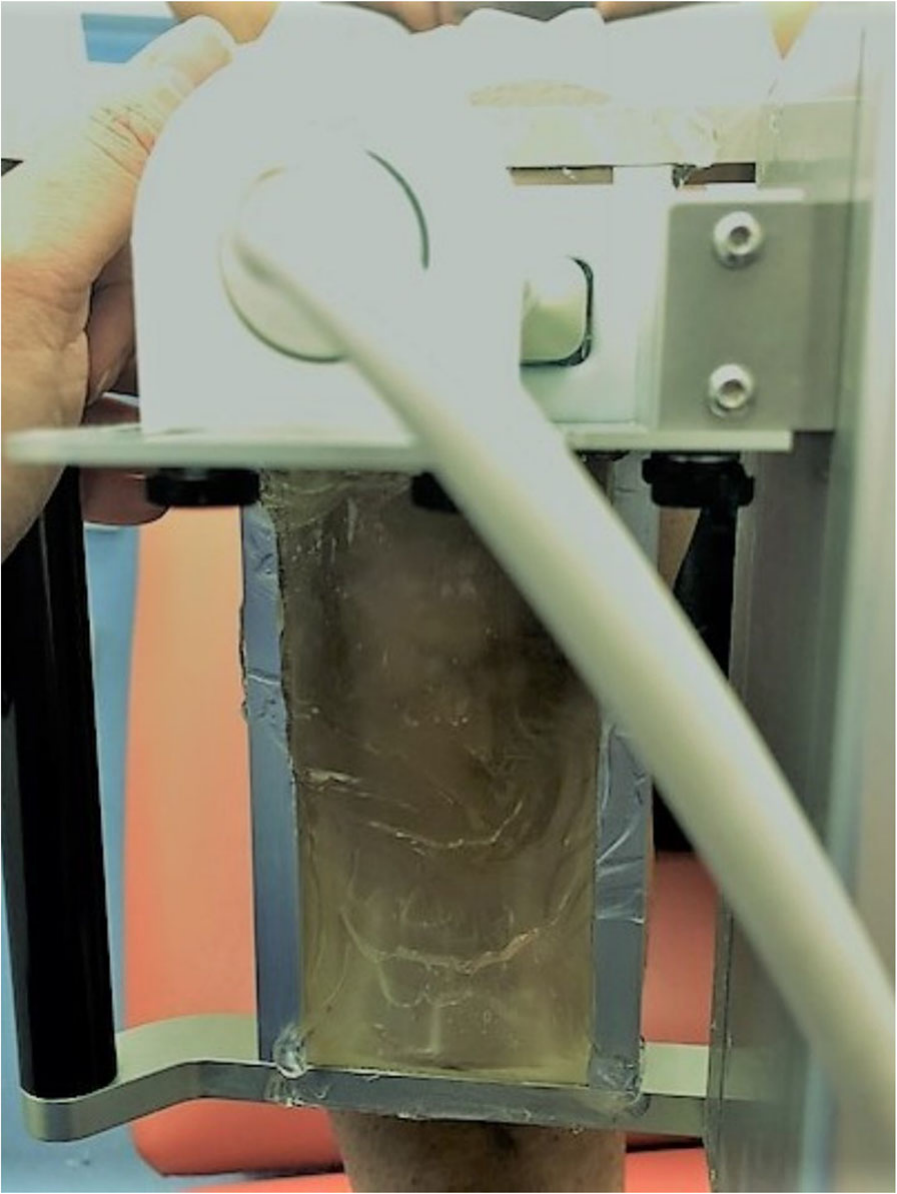
Superior view of UTC transducer transversely fixed into tracking device for scanning a right patellar tendon

**Fig. 2:**
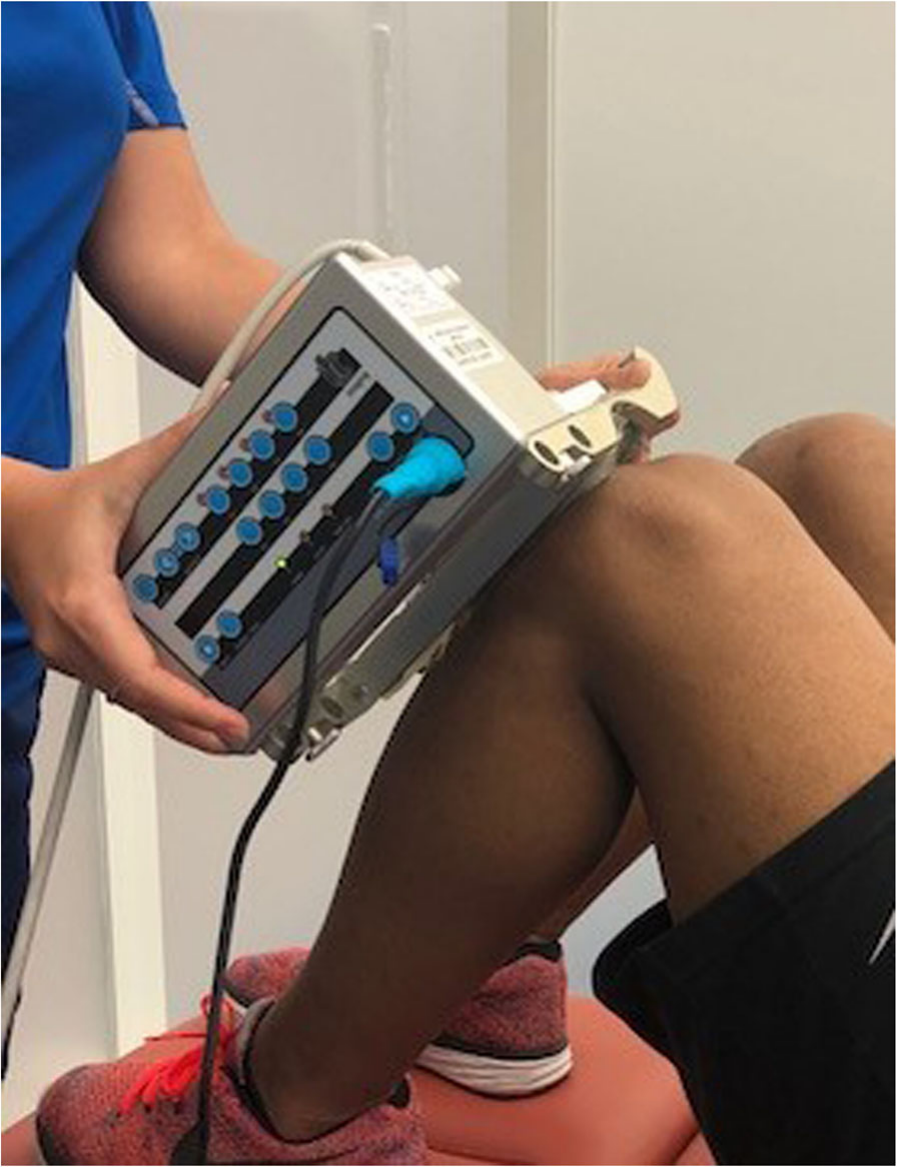
Lateral view of UTC tracking device showing silicone pad in contact with left patellar tendon

**Fig. 3:**
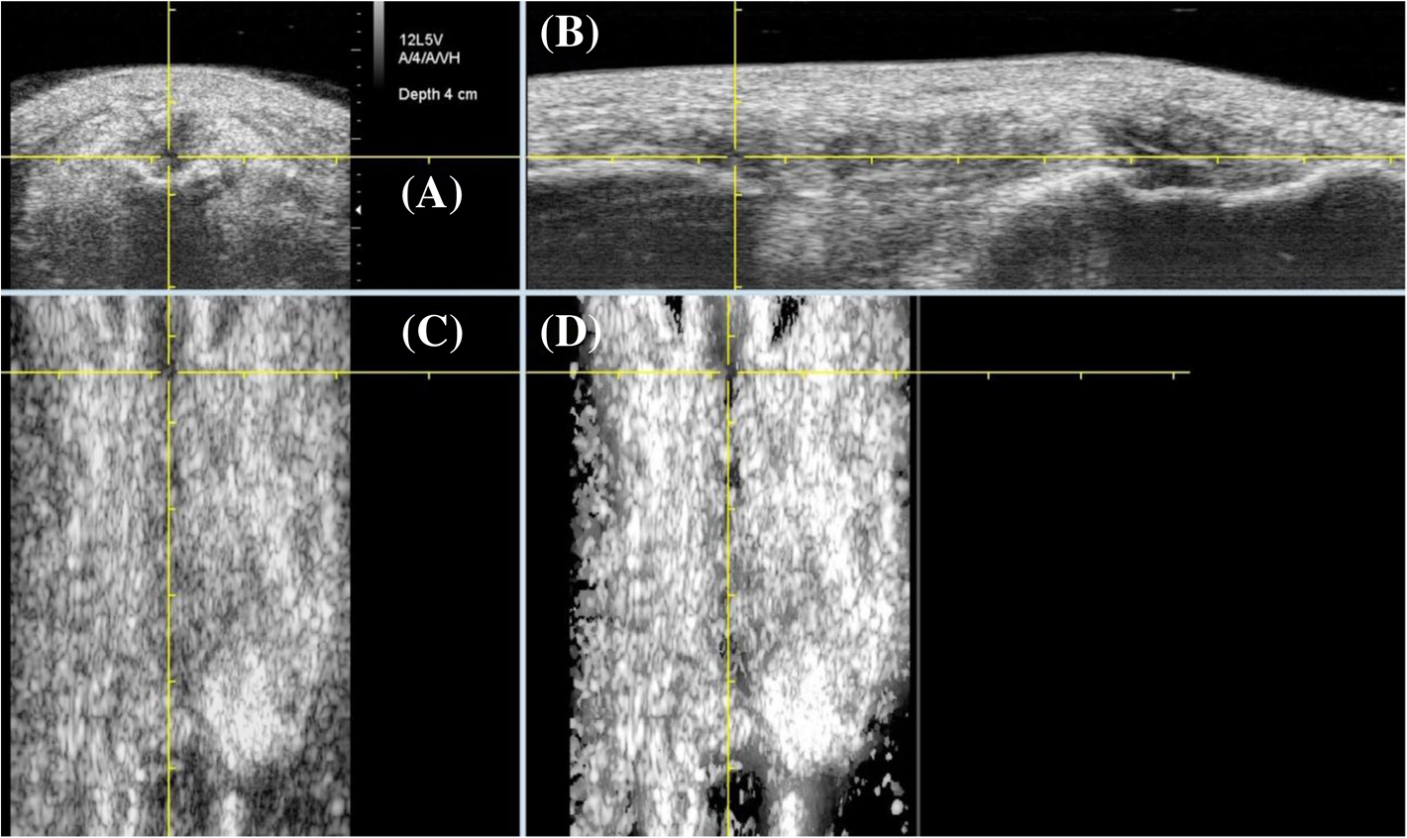
Transverse (**a**), sagittal (**b**), and coronal (**c** & **d**) views of a harvested patellar tendon. The cross-hair is placed in the center of the harvested region (3A) at the distal pole of the patella (3B). Horizontal line ensures that patella and tibia tuberosity are at the same level (3B). The vertical lines in (3C) and (3D) allow confirmation that patella apex and tibia tuberosity are aligned. Horizontal and vertical alignment are requirements for a scan to be considered of a satisfactory quality to be saved and included for analysis

UTC imaging utilizes a 5–12 MHz ultrasound (US) transducer (SmartProbe 12 L5, Terason 2000, Teratech, USA) fixed in a transverse position into a 12 cm tracking device (UTC Tracker, UTC imaging, Netherlands), allowing the capture and storage of a sequence of transverse images of the tendon at regular intervals of 0.02 cm (Fig. 1). Participants lay supine with their knees flexed at approximately 100° and their feet parallel resting on the plinth. Coupling gel was applied between the US probe and the stand-off pad, and between the stand-off pad and the skin to optimize contact. The examiners held the UTC tracker device resting with full contact on participant’s anterior knee, parallel to the long axis of the patellar tendon (Fig. 2). The US transducer was placed initially over the apex of the patella and manually moved down to ensure the patellar tendon was centrally located on the transverse view in the UTC acquisition software. Once a good position was visually affirmed, the data acquisition was initiated. The US transducer then moved down the track driven by a motor, from proximal to distal, resulting in a total of 598 sequential transverse images acquired in 45 s. With these scans the UTC algorithm creates a 3D block of the scanned area allowing additional reconstructed coronal and sagittal views (Fig. 3). A scan was considered satisfactory and included for analysis when the upper surface of the patella and tibial tuberosity were at the same level with the patellar tendon horizontal and taut on the sagittal view of the UTC acquisition software, and the patella and tibial tuberosity were aligned longitudinally with the patellar tendon vertically displayed in the coronal view of the UTC acquisition software (Fig. 3). The patellar tendons of participants whose UTC scans did not meet the above criteria (mostly due to painful limited knee flexion post-operatively) were excluded. Due to the presence of swelling and thickness of the harvested patellar tendon we adopted the factory preset of the UTC imaging software for patellar tendons (PT_UTC_VH4028) for medium size participants, with US parameters standardized as: 12 MHz, focus at 2.8 cm, and depth of 4 cm. For these settings each pixel unit can be considered as equivalent to 1.0 mm. In all cases the right knee was scanned first.

### UTC data analysis and processing

**Fig. 4:**
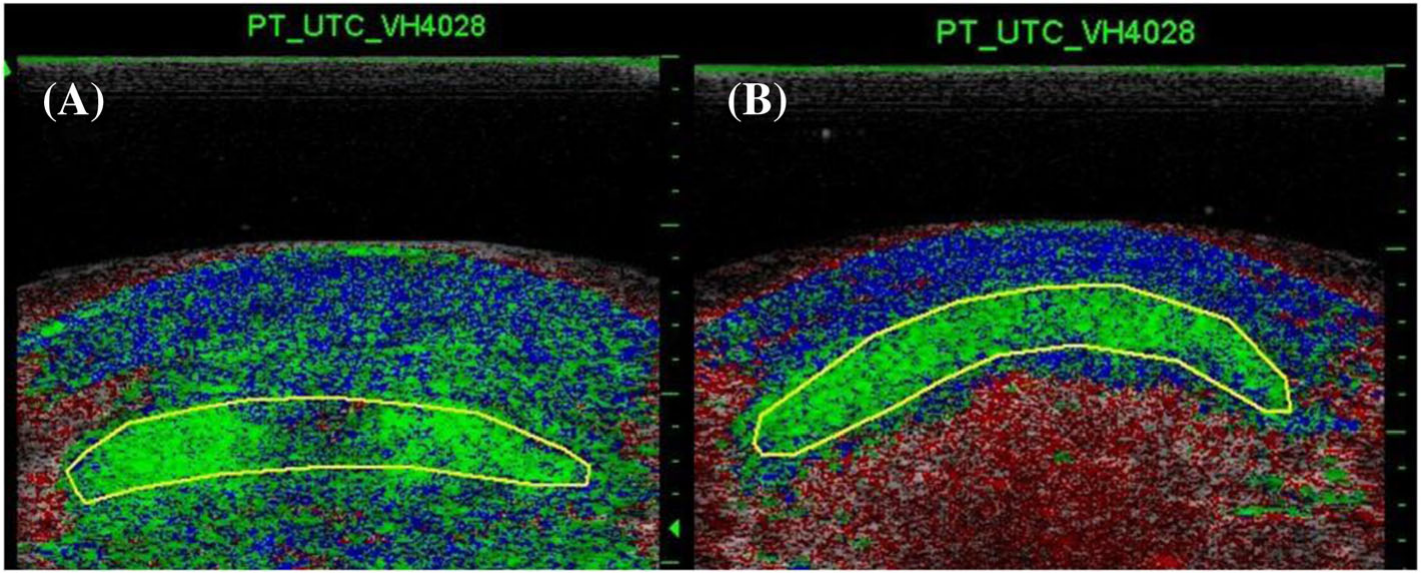
Example of contours drawn in cross-sectional view in harvested **(a)** and unharvested **(b)** patellar tendons. Echo-types I are shown as green, echo-type 2 as blue, echo-type III as red, and echo-type 4 as black. Note that only the area inside the marked yellow circumference is quantified as patellar tendon, and it is in this area that all calculations regarding relative percentages of different echo-types are made

**Fig. 5:**
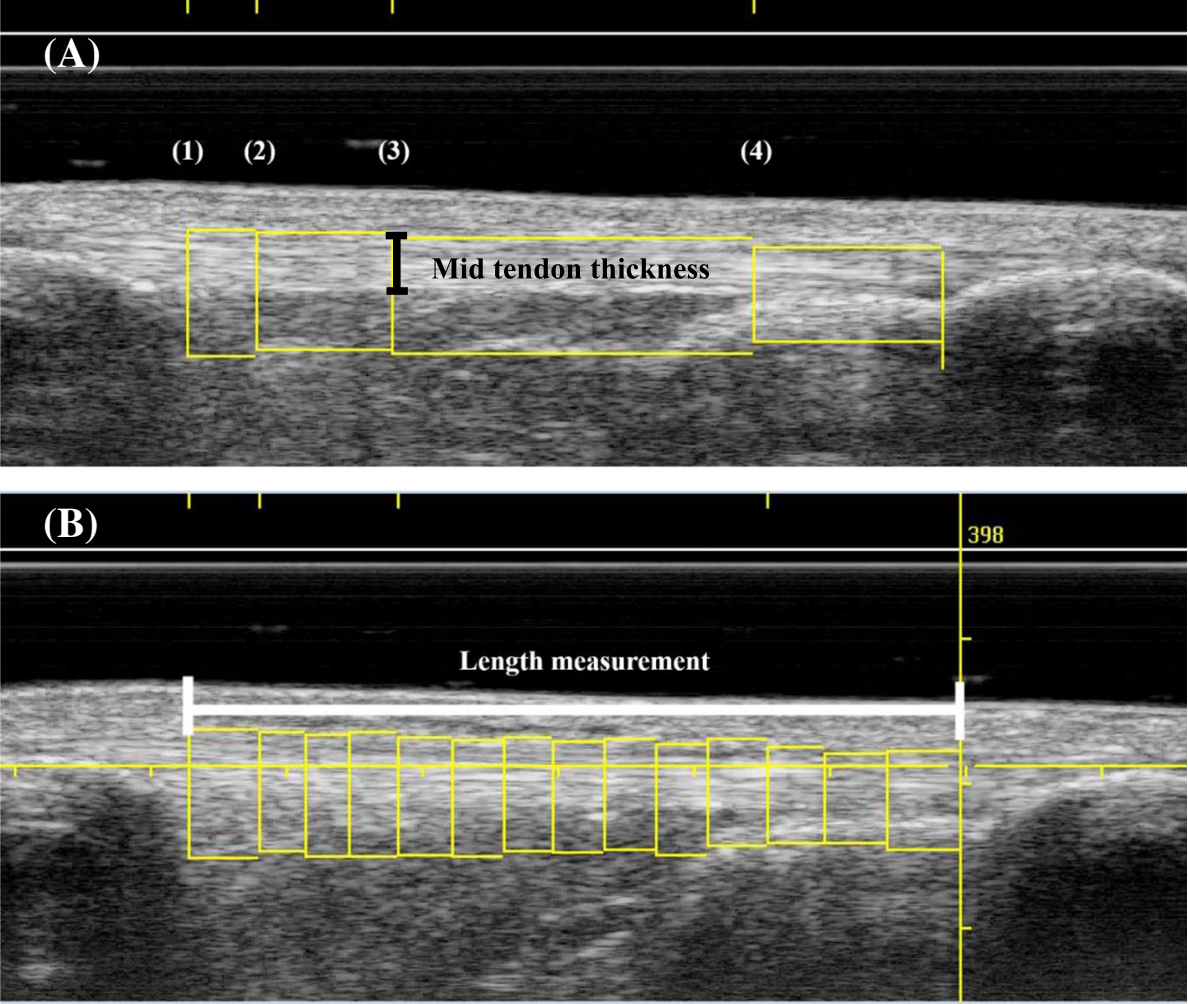
Example of sagittal images of the patellar tendon after acquisition with UTC imaging. **a** The four areas of interest (1–4) are depicted. The vertical black bar shows the measurement of tendon thickness. The first area of interest (patella apex) was defined as the first image distal to the patella apex. The second area of interest (proximal tendon) started 0.52 cm distal to mark 1, and the third (mid tendon) 1.54 cm further distally. The fourth area of interest (distal tendon) was set at 75% of tendon length. **b** The length of the tendon is depicted by the horizontal white bar. In this case, 15 contours were drawn as shown. In all subjects a minimum of 10 of such contours were created. Each of these contours were individually marked in a transverse view to outline the extent of the tendon (see Fig. 4) for that area of interest

All analyses were performed on the UTC analyzer v.2.0.2 using a window size 17. Two examiners scanned the same patellar tendons on the same day. Only one examiner scanned the same patellar tendons twice, 1 day apart. Subsequently software analysis of the same patellar tendons was performed on different days to avoid any possibility of bias in this phase. For the analysis, the margin of the patellar tendon (contour) was manually traced in the transverse images of the tendon creating at least 10 sections along the patellar tendon length to quantify the whole tendon structure (Fig. 4). The first contour of each tendon was drawn from the notch of the tibia. This contour determines the last (most distal) transverse image included in the patellar tendon characterization analysis. The examiner ensured longitudinal alignment between the notch of the tibia and the patellar apex to draw this contour. The second contour was drawn from the first transverse image immediately distal to the patellar apex. This is the first area of interest, set as reference mark 1 in the UTC acquisition software, and is the first transverse image included in the characterization analysis which defines the beginning of the patellar tendon length measurement. Twenty-six images distal to reference mark 1, another contour was drawn (2nd area of interest = reference mark 2) representing the proximal area of the patellar tendon (0.52 cm distal from patellar apex). The 3rd area of interest or mid tendon (reference mark 3) was drawn 51 images distal from reference mark 2 (1.54 cm distal from patellar apex). [8, 26] Additionally, at 75% of the distance between the reference mark 1 and the notch of the tibia (last contour), a fourth contour was drawn (4th area of interest = reference mark 4) to characterize the distal part of the patellar tendon (Fig. 5a). Between reference marks 2 and 3, another two contours were drawn approximately 0.5 cm apart. Between reference marks 3 and 4, additional contours were drawn at approximately 0.5 cm intervals, and between reference mark 4 and the notch of the tibia another contour was drawn. (Note that each additional contour provided to the software reduces the amount of interpolation required to depict the patellar tendon.) Only the transverse images between the patellar apex and the notch of the tibia were considered in the characterization analysis of the patellar tendon. Measurement of thickness of the mid tendon was done manually using the measuring tool of the UTC imaging software (Fig. 5a). The distance in centimeters between the first and last contours represents the length of the patellar tendon (Fig. 5b).

The UTC algorithm quantifies the proportion of echo-types in each specific area of interest, (1) patellar apex, (2) proximal tendon, (3) mid tendon, (4) distal tendon, and (5) overall tendon (all the tendon information between the first and last contours, patellar apex and notch of the tibia, respectively). Four sub-types of tendon are classified according to 4 primary tendon features appearing on grayscale ultrasound images: continuity, integrity and alignment of the collagen tendon bundles, and brightness [2]. In essence, alignment is measured by the degree of variation from a true, straight line of a series of pixels within the window being examined. Variation in brightness is estimated by comparing adjacent pixels on their grayscale value – i.e. the representation of the pixel on a scale from complete black through to bright white. The echo-type I (green) is generated by intact and aligned collagen bundles. These collagen bundles appear linear within the window, with little to no variation in their grayscale “whiteness” value. The echo-type II (blue) is reported in the presence of discontinuous, swollen, and wavy collagen bundles. It is defined by pixels that are aligned but display variation of about 10% of the gray levels. The echo-type III (red) is generated by a loose matrix consisting mainly of smaller fibrils. It is represented by much less aligned pixels with gray level variation of more than 10%. The echo-type IV (black) is generated by mainly amorphous matrix with loose fibrils, cells and fluid (hematoma and exudate). It is represented by echoes with a severe lack of stability and no pixels alignment over sequential transverse images. [2, 3] In addition to echo-type characterization, the UTC algorithm also quantifies the area within the contour (volume) drawn in four selected areas of interested (reference marks 1–4).

After running the UTC software analyses, a range of raw data was exported for analysis.

The following variables were assessed: length of the patellar tendon; thickness of the mid tendon; percentage of echo-types I, II, III, and IV; tendon volume at patellar apex, proximal, mid, and distal tendon; and percentage of echo-types I, II, III and IV in the whole tendon.

### Repeated measures - intra-rater and inter-rater reliabilities

**Fig. 6:**
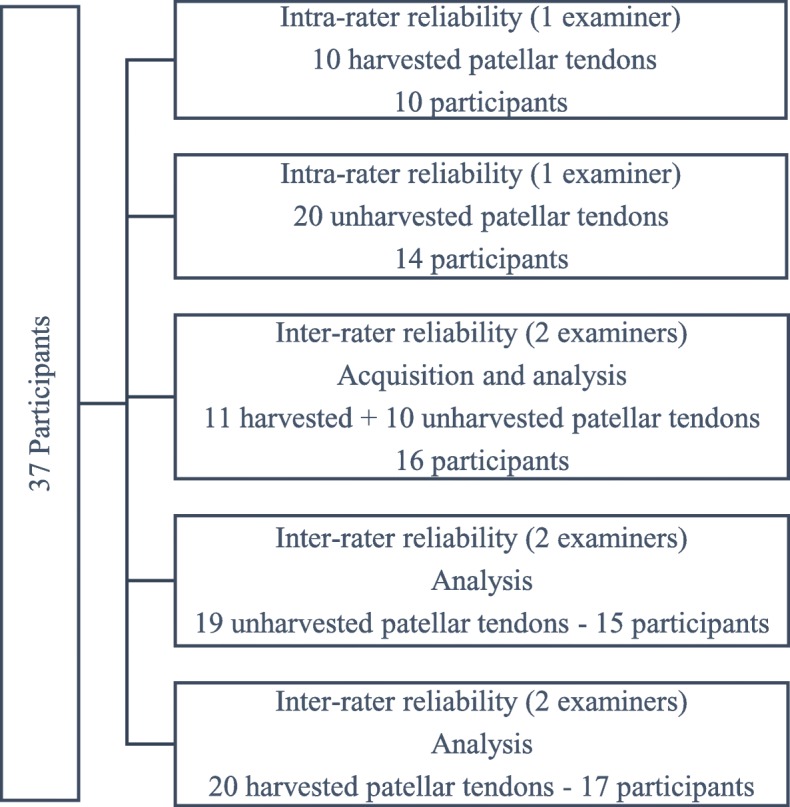
Description of the number of patellar tendons and participants included, and the analyses performed

Twenty unharvested and ten harvested patellar tendons from 18 participants were scanned and analyzed twice, 1 day apart, by the same examiner (C.S.P., physiotherapist – 3 years of experience with UTC imaging acquisition and analysis) to test the intra-rater reliability of acquisition and analysis. Ten unharvested and eleven harvested (ACLR) patellar tendons from 16 participants were scanned, on the same day, by two different examiners (C.S.P. and R.C.G.S., sports physician – 6 months of experience with UTC imaging acquisition and analysis) to investigate inter-rater reliability. For these tendons, each examiner analyzed their own scans to test the inter-rater reliability of the acquisition and the analysis. Additionally, nineteen unharvested and twenty harvested patellar tendons from 23 participants were analyzed by the two examiners (C.S.P. and R.C.G.S) to describe the inter-rater reliability of the analysis (Fig. 6).

### Statistical analysis

Descriptive statistics (mean ± standard deviation) were calculated for participants’ demographics and all UTC variables. Data was tested for normality through visual inspection of histograms and Q-Q plots as well as calculation of Shapiro-Wilk statistics. The majority of the studied variables in harvested and unharvested tendons were normally distributed with exception of the following 9 variables: tendon thickness, percentage of echo-types III at proximal tendon, and percentage of echo-types III and IV at the distal tendon in harvested tendons, and the variables of percentage of echo-types I and II at mid-tendon, and percentage of echo-types III and IV in all areas of unharvested tendons. [27]

Test – retest reliability of both UTC data collection and analysis were assessed for harvested and unharvested patellar tendons. Two-way mixed single measures intra-class correlation for absolute agreement between repeated scans (ICC_2,1_) was calculated to yield the standard error of the measurement (SEM = SD (Day 1) × [√ (1-ICC)]), [17, 28] standard error of measurement as percentage of the grand mean (SEM % GrM = SEM/ Average Acquisitions 1&2 × 100), and the minimal detectable change of all UTC parameters (MDC = 1.96 × SEM × √2). [7, 14, 16, 18, 29, 30] MDC for the variables of harvested and unharvested patellar tendons were calculated based on the intra-rater reliability analysis, when tendon scanning and contour drawing were performed two times by the same examiner (C.S.P.). ICC values were considered poor when less than 0.40, fair between 0.40 and 0.59, good between 0.60 and 0.74, and excellent when above 0.75. [3, 31] 95% confidence intervals (CI) are reported parenthetically after the group estimator where applicable. SPSS version 21 was used for all statistical analyses (SPSS Inc., Chicago, Illinois, USA).

## Results

The mean age of the participants at the time of data acquisition was 23 years (range: 16 to 36 years), body mass of 75.9 ± 15 kg, and height of 177 ± 11 cm. The sport, the time of the data acquisition, and the type of graft used for the ACLR for each participant are detailed in Table 1.

### Repeated measures – intra-rater reliability

Analysis to quantify the proportion of each of the echo-types (I, II, III and IV) in each of the areas of interest (patellar apex, proximal tendon, mid tendon, distal tendon, and overall tendon) of harvested and unharvested patellar tendons displayed excellent intra-rater reliability (ICC_2,1_: 0.95–0.99 harvested, 0.89–0.98 unharvested) (Table 2). Intra-rater reliability for the measure of volume in the four areas of interest of the tendon was good (ICC_2,1_: 0.69 harvested, 0.67 unharvested), and the intra-rater reliability for the measure of thickness of the mid tendon was excellent for harvested (ICC_2,1_: 0.88) but fair for unharvested (ICC_2,1_: 0.57) tendons (Table 3). The measurement of tendon length displayed excellent intra-rater reliability (4.5 ± 0.6 cm, ICC_2,1_ = 0.79, SEM = 0.3 cm, SEM % GrM = 7.4%, MDC = 0.9 cm) for harvested tendons, and (4.9 ± 0.7 cm, ICC_2,1_ = 0.94, SEM = 0.2 cm, SEM % GrM = 3.6%, MDC = 0.5 cm) unharvested tendons.

**Table 2:** Echo-types values of harvested and unharvested tendons for the two acquisition days, done by one examiner

Intra-rater reliability for acquisition and analysis – 1 examiner / 2 acquisitions/ 2 different days/ 2 analysis per patellar tendon											
Examiner C.S.P.	DAY 1				DAY 2						
Area/ Echo-types	Type I (%)	Type II (%)	Type III (%)	Type IV (%)	Type I (%)	Type II (%)	Type III (%)	Type IV (%)	ICC (95% CI)	SEM (ICC) % GrM (%)	SEM (ICC) (%)
Harvested patellar tendons of 10 participants (*n* = 10)											
Patellar Apex	56.0 ± 10.8	33.5 ± 5.9	7.3 ± 4.8	2.9 ± 1.6	54.7 ± 9.8	31.6 ± 6.9	8.9 ± 4.2	4.8 ± 2.6	0.95 (0.89–0.97)	21.1	5.3
Proximal tendon	60.0 ± 6.6	32.1 ± 5.1	5.8 ± 3.9	2.2 ± 1.5	60.5 ± 8.8	29.4 ± 6.3	6.6 ± 4.1	3.6 ± 2.8	0.97 (0.94–0.98)	16.1	4.0
Mid Tendon	62.8 ± 7.0	29.4 ± 4.0	5.4 ± 3.5	2.5 ± 1.7	64.8 ± 8.8	27.2 ± 6.8	5.2 ± 3.3	3.1 ± 2.6	0.98 (0.95–0.98)	15.1	3.8
Distal Tendon	46.9 ± 7.9	35.3 ± 6.3	13.2 ± 8.4	4.5 ± 2.6	50.0 ± 12.6	34.0 ± 7.6	11.4 ± 5.8	4.6 ± 2.8	0.95 (0.89–0.97)	17.2	4.3
Overall Tendon	53.6 ± 5.4	33.3 ± 4.1	9.5 ± 4.7	3.7 ± 1.7	54.9 ± 7.0	31.6 ± 4.5	9.2 ± 4.3	4.1 ± 2.4	0.99 (0.97–0.99)	9.0	2.2
Unharvested patellar tendons of 14 participants (*n* = 20)											
Patellar Apex	57.5 ± 12.3	39.0 ± 10.6	2.5 ± 5.2	0.9 ± 1.9	56.1 ± 13.3	40.5 ± 12.0	2.6 ± 4.7	0.8 ± 1.6	0.94 (0.90–0.96)	25.5	6.4
Proximal tendon	65.7 ± 10.3	33.0 ± 8.6	0.8 ± 2.3	0.3 ± 0.9	67.4 ± 8.5	31.9 ± 8.0	0.5 ± 0.9	0.2 ± 0.4	0.97 (0.94–0.97)	21.0	5.2
Mid Tendon	68.6 ± 10.2	30.1 ± 8.7	0.9 ± 1.9	0.3 ± 0.7	70.2 ± 9.3	29.0 ± 8.6	0.4 ± 1.2	0.2 ± 0.5	0.98 (0.97–0.98)	14.6	3.7
Distal Tendon	53.3 ± 10.1	41.3 ± 7.8	4.3 ± 5.6	1.2 ± 2.0	52.9 ± 17.7	42.2 ± 12.9	4.1 ± 9.3	0.7 ± 1.7	0.89 (0.83–0.92)	31.6	7.9
Overall Tendon	60.4 ± 7.8	35.2 ± 6.3	3.2 ± 3.3	0.8 ± 1.2	61.6 ± 9.8	33.9 ± 6.9	3.6 ± 4.3	0.8 ± 1.2	0.98 (0.96–0.98)	15.1	3.8

*n* = number of tendons assessed. Mean ± standard deviation

*ICC (95% CI)* Intra-class coefficient of reliability (95% confidence interval), *SEM % GrM* Standard error of measurement as percentage of the grand mean, *SEM* Standard error of measurement

**Table 3:** Values of volume and thickness of harvested and unharvested tendons over two acquisition days, one examiner

Intra-rater reliability for acquisition and analysis – 1 examiner / 2 acquisitions/ 2 different days/ 2 analysis per patellar tendon				
24 Participants				
Examiner C.S.P.	Harvested patellar tendon (*n* = 10)		Unharvested patellar tendon (*n* = 20)	
Areas of interest	Volume (cm^3^)	Thickness (cm)	Volume (cm^3^)	Thickness (cm)
DAY 1				
Patellar Apex	1.4 ± 0.3	NA	1.0 ± 0.2	NA
Proximal tendon	1.3 ± 0.2	NA	0.9 ± 0.1	NA
Mid Tendon	1.3 ± 0.2	0.7 ± 0.2	0.9 ± 0.2	0.5 ± 0.1
Distal Tendon	1.2 ± 0.2	NA	0.8 ± 0.1	NA
DAY 2				
Patellar Apex	1.4 ± 0.2	NA	1.0 ± 0.2	NA
Proximal tendon	1.3 ± 0.2	NA	1.0 ± 0.2	NA
Mid Tendon	1.2 ± 0.2	0.7 ± 0.2	0.9 ± 0.2	0.5 ± 0.1
Distal Tendon	1.3 ± 0.2	NA	0.9 ± 0.1	NA
ICC (95% CI)	0.69 (0.48, −0.82)	0.88 (0.60, − 0.96)	0.67 (0.52, − 0.77)	0.57 (0.20, 0.80)
SEM (ICC) % GrM	10.6	8.9	10.2	8.7
SEM (ICC)	0.1	0.1	0.1	0.04
MDC	0.4	0.2	0.3	0.1

*n* = number of tendons assessed. Mean ± standard deviation

*ICC (95% CI)* Intra-class coefficient of reliability (95% confidence interval), *SEM % GrM* Standard error of measurement as percentage of the grand mean, *SEM* Standard error of measurement, *MDC* Minimal detectable change

The minimal detectable change for harvested tendons was 7.5% for echo-type I, 6.9% for echo-type II, 4.8% for echo-type III and 2% for echo-type IV. For unharvested tendons, the MDC was 14.1% for echo-type I, 10.6% for echo-type II, 6.3% for echo-type III and 1.2% for echo-type IV.

### Repeated measures – inter-rater reliability – acquisition and analysis

Analysis of the amount of echo-types I, II, III and IV in the four areas of interest and in the overall tendon when two examiners acquired and analyzed their own scans of mixed harvested and unharvested patellar tendons demonstrated excellent inter-rater reliability (ICC_2,1_: 0.89–0.98) (Table 4). Volume of the tendon in the areas of interest, and thickness of the mid tendon showed good (ICC_2,1_: 0.67) and excellent (ICC_2,1_: 0.97) inter-rater reliability, respectively (Table 5). The tendon length of mixed harvested and unharvested tendons appeared to have good inter-rater reliability (4.5 ± 0.5 cm, ICC_2,1_ = 0.63, SEM = 0.2 cm, SEM % GrM = 7.3%).

**Table 4:** Echo-types values in harvested and unharvested tendons – performed by two examiners, each examiner taking two acquisitions on the same day

Inter-rater reliability for acquisition and analysis - 2 acquisitions/ same day/ 2 examiners/ 2 analysis per patellar tendon											
Harvested and Unharvested patellar tendons of 16 participants - (*n* = 21)											
Area/ Echo-types	Examiner 1 (C.S.P.)				Examiner 2 (R.C.G.S.)						
	Type I (%)	Type II (%)	Type III (%)	Type IV (%)	Type I (%)	Type II (%)	Type III (%)	Type IV (%)	ICC (95% CI)	SEM (ICC) % GrM (%)	SEM (ICC) (%)
Patellar Apex	55.4 ± 13.2	31.0 ± 8.2	8.8 ± 8.5	4.8 ± 5.0	49.0 ± 12.6	36.3 ± 8.5	10.2 ± 10.5	4.5 ± 5.0	0.94 (0.90, − 0.96)	25.5	6.4
Proximal tendon	60.8 ± 15.4	27.0 ± 8.8	7.7 ± 8.4	4.6 ± 5.3	55.8 ± 16.4	30.5 ± 6.1	9.8 ± 12.4	4.0 ± 4.9	0.97 (0.94, − 0.97)	21.0	5.2
Mid Tendon	60.6 ± 17.5	26.4 ± 9.3	8.5 ± 10.1	4.5 ± 4.6	58.7 ± 14.1	29.2 ± 7.0	8.1 ± 9.0	4.0 ± 5.8	0.98 (0.97, − 0.98)	14.6	3.7
Distal Tendon	52.2 ± 16.6	26.2 ± 9.6	14.0 ± 10.3	7.4 ± 6.0	55.8 ± 16.5	28.1 ± 6.3	11.1 ± 12.1	5.2 ± 4.6	0.89 (0.83, − 0.92)	31.6	7.9
Overall Tendon	54.2 ± 15.6	25.4 ± 7.9	13.4 ± 9.6	7.1 ± 5.4	53.1 ± 13.9	29.2 ± 4.9	12.3 ± 9.4	5.6 ± 4.4	0.98 (0.96, − 0.98)	15.1	3.8

*n* = number of tendons assessed. Mean ± standard deviation

*ICC (95% CI)* Intra-class coefficient of reliability (95% confidence interval), *SEM % GrM* Standard error of measurement as percentage of the grand mean, *SEM* Standard error of measurement

**Table 5:** Volume and thickness for harvested and unharvested tendons - two examiners taking two acquisitions on the same day

Inter-rater reliability for acquisition and analysis - 2 examiners/ 2 acquisitions/ same day/ 2 analysis per tendon		
6 Participants		
Harvested (*n* = 11) and Unharvested (*n* = 10) patellar tendons		
Areas of interest	Volume (cm^3^)	Thickness (cm)
Examiner1 (C.S.P.)		
Patellar Apex	1.1 ± 0.4	NA
Proximal tendon	1.1 ± 0.4	NA
Mid Tendon	1.0 ± 0.3	0.6 ± 0.3
Distal Tendon	1.0 ± 0.4	NA
Examiner 2 (R.C.G.S.)		
Patellar Apex	0.9 ± 0.2	NA
Proximal tendon	1.0 ± 0.2	NA
Mid Tendon	1.0 ± 0.3	0.6 ± 0.2
Distal Tendon	1.0 ± 0.3	NA
ICC (95% CI)	0.67 (0.52, −0.77)	0.97 (0.91, − 0.98)
SEM (ICC) % GrM	10.2	7.8
SEM (ICC)	0.1	0.05

*n* = number of tendons assessed. Mean ± standard deviation

*ICC (95% CI)* Intra-class coefficient of reliability (95% confidence interval), *SEM % GrM* Standard error of measurement as percentage of the grand mean, *SEM* Standard error of measurement

### Repeated measures – inter-rater reliability - analysis

When two examiners analyzed the same scan of harvested and unharvested tendons separately, the inter-rater reliability was excellent for the echo-type variables in the different areas of interest (ICC_2,1_: 0.95–0.99) (Table 6) and mid tendon thickness of harvested tendons (ICC_2,1_: 0.85) (Table 7). The inter-rater reliability of the volume in different levels of the tendon was fair for harvested (ICC_2,1_: 0.59) and poor for unharvested (ICC_2,1_: 0.30) tendons (Table 7). Moreover, the mid tendon thickness of unharvested tendons also displayed poor inter-rater reliability (ICC_2,1_: 0.24) when two examiners analyzed the same scan (Table 7). On the other hand, tendon length displayed excellent reliability for both harvested and unharvested tendons (4.7 ± 0.7 cm, ICC_2,1_ = 0.86, SEM = 0.3 cm, SEM % GrM = 5.4%, and 4.8 ± 0.6 cm, ICC_2,1_ = 0.79, SEM = 0.3 cm, SEM %GrM = 6.6% respectively).

**Table 6:** Echo-type values in harvested and unharvested tendons – one acquisition made by one examiner with two examiners analyzing (the same scan data)

Inter-rater reliability of analysis - 1 acquisition/ 1 day/ 2 examiners/ 2 analysis per patellar tendon											
Area/ Echo-types	Examiner 1 (C.S.P.)				Examiner 2 (R.C.G.S.)						
	Type I (%)	Type II (%)	Type III (%)	Type IV (%)	Type I (%)	Type II (%)	Type III (%)	Type IV (%)	ICC (95% CI)	SEM (ICC) % GrM (%)	SEM (ICC) (%)
Harvested patellar tendons of 17 participants (*n* = 20)											
Patellar Apex	52.8 ± 10.0	31.3 ± 6.3	10.4 ± 5.9	5.4 ± 3.5	52.0 ± 10.0	32.3 ± 6.9	10.5 ± 5.8	5.3 ± 3.0	0.98 (0.97, − 0.98)	10.5	2.6
Proximal tendon	57.4 ± 8.8	29.4 ± 4.8	8.7 ± 5.8	4.7 ± 4.0	57.3 ± 7.9	29.3 ± 5.0	8.6 ± 4.8	4.8 ± 3.2	0.99 (0.97, − 0.99)	10.4	2.6
Mid Tendon	58.5 ± 10.1	28.1 ± 4.8	8.8 ± 6.2	5.0 ± 3.9	58.5 ± 10.8	29.0 ± 7.3	7.8 ± 6.8	4.9 ± 4.7	0.98 (0.97, − 0.98)	11.3	2.8
Distal Tendon	47.1 ± 8.7	29.3 ± 8.6	15.7 ± 8.0	7.9 ± 5.2	48.8 ± 11.1	30.5 ± 9.1	13.8 ± 7.7	7.1 ± 5.1	0.95 (0.92, − 0.96)	15.2	3.8
Overall Tendon	50.7 ± 7.3	28.7 ± 5.8	13.7 ± 6.6	7.1 ± 4.5	51.2 ± 6.0	29.9 ± 5.5	12.6 ± 5.6	6.6 ± 4.0	0.99 (0.98, − 0.99)	6.8	1.7
Unharvested patella tendons of 15 participants (*n* = 19)											
Patellar Apex	62.2 ± 6.7	36.6 ± 6.2	0.8 ± 1.2	0.3 ± 0.5	60.9 ± 11.3	37.3 ± 10.4	1.4 ± 2.1	0.4 ± 1.0	0.97 (0.94–0.97)	19.1	4.8
Proximal tendon	70.2 ± 5.6	29.0 ± 5.5	0.4 ± 0.8	0.2 ± 0.4	68.3 ± 6.9	30.3 ± 6.3	0.9 ± 1.3	0.3 ± 0.5	0.99 (0.98–0.99)	12.8	3.2
Mid Tendon	72.6 ± 6.3	26.5 ± 6.6	0.5 ± 0.8	0.2 ± 0.5	70.7 ± 5.5	27.4 ± 5.2	1.0 ± 1.7	0.6 ± 1.2	0.99 (0.97–0.99)	13.7	3.4
Distal Tendon	58.3 ± 10.9	36.8 ± 9.4	3.7 ± 3.9	1.3 ± 1.5	57.9 ± 10.0	37.6 ± 6.7	3.3 ± 4.7	1.2 ± 1.9	0.95 (0.91–0.96)	23.0	5.8
Overall Tendon	64.3 ± 5.4	31.4 ± 5.4	2.9 ± 2.4	1.1 ± 1.2	63.3 ± 4.4	33.0 ± 4.1	2.8 ± 2.5	1.1 ± 1.1	0.99 (0.98–0.99)	9.4	2.3

*n* = number of tendons assessed. Mean ± standard deviation

*ICC (95% CI)* Intra-class coefficient of reliability (95% confidence interval), *SEM % GrM* Standard error of measurement as percentage of the grand mean, *SEM* Standard error of measurement

**Table 7:** Values of volume and thickness in harvested and unharvested patellar tendons – one acquisition, two examiners

Inter-rater reliability of analysis - 1 acquisition/ 1 day/ 2 examiners/ 2 analysis per patellar tendon				
23 Participants				
Areas of interest	Harvested patellar tendons (*n* = 20)		Unharvested patellar tendons (*n* = 19)	
	Volume (cm^3^)	Thickness (cm)	Volume (cm^3^)	Thickness (cm)
Examiner 1 (C.S.P.)				
Patellar Apex	1.5 ± 0.3	NA	0.9 ± 0.2	NA
Proximal tendon	1.4 ± 0.4	NA	0.9 ± 0.1	NA
Mid Tendon	1.3 ± 0.3	0.7 ± 0.2	0.9 ± 0.1	0.4 ± 0.1
Distal Tendon	1.3 ± 0.3	NA	0.8 ± 0.2	NA
Examiner 2 (R.C.G.S.)				
Patellar Apex	1.2 ± 0.3	NA	0.9 ± 0.2	NA
Proximal tendon	1.3 ± 0.3	NA	0.9 ± 0.2	NA
Mid Tendon	1.4 ± 0.3	0.7 ± 0.2	1.0 ± 0.3	0.4 ± 0.0
Distal Tendon	1.4 ± 0.2	NA	0.9 ± 0.2	NA
ICC (95% CI)	0.59 (0.42, −0.71)	0.85 (0.66, 0.94)	0.30 (0.08, −0.49)	0.24 (− 0.20, 0.62)
SEM (ICC) % GrM	16.4	13.9	14.6	13.5
SEM (ICC)	0.2	0.1	0.1	0.1

*n* = number of tendons assessed. Mean ± standard deviation

*ICC (95% CI)* Intra-class coefficient of reliability (95% confidence interval), *SEM % GrM* Standard error of measurement as percentage of the grand mean, *SEM* Standard error of measurement

## Discussion

This is the first study to investigate the reliability of UTC in the measurement of tendon structure following ACLR. Results of the current study suggests that the UTC imaging displays excellent reliability for quantifying the proportion of each of the echo-types (I, II, III and IV) in each of the areas of interest (patellar apex, proximal tendon, mid tendon, distal tendon, and overall tendon) and mid tendon thickness, and fair to good reliability for the measure of volume in all areas of interest of harvested patellar tendons. For unharvested patellar tendons, results suggest excellent reliability for the distribution of the four echo-types in all areas of interest, poor to good reliability for volume in the four selected areas, and poor to fair reliability for mid tendon thickness. Consequently, UTC may be a useful tool to characterize the quality of harvested patellar tendons after ACLR at different time points.

Results of the current study are in agreement with previous studies of normal and pathological Achilles [3, 7, 18] and patellar tendons [10, 26, 32], where excellent intra-rater reliability was found for the echo-types variables. Regardless of the examiners acquiring and analyzing different scans or different examiners analyzing the same scan, the intra- and inter-rater reliability for all the four echo-types in all areas of interest displayed excellent reliability for harvested and unharvested tendons, as well as mid tendon thickness of harvested patellar tendons and tendon length.

The reliability of the measure of mid tendon thickness (ICC _2,1_: 0.85–0.88, 0.7 cm harvested, 0.4–0.5 cm unharvested patellar tendons) compares favorably with the measurements of patellar tendon thickness observed by Hernandez et al., [26] who reported thickness of 0.5 cm at the mid tendon of basketball players, and with the Achilles tendon thickness observed by van Schie et al. [3] who reported reliability values of ICC = 0.84 and measurements of “anterior-posterior diameter” of 0.9 cm for symptomatic and 0.7 cm for asymptomatic Achilles tendons. However, despite the similar mean values obtained by each examiner, the very small standard error of measurement, and the comparable values with healthy patellar tendon thickness measured in previous studies [33, 34], the mid tendon thickness of unharvested tendons displayed poor inter-rater reliability. We suspect that this error may arise due to the precision of the measuring tool of the UTC imaging software. Specifically, this tool only reports to an accuracy of 0.1 cm within the 3-D constructed tendon block, and nearly all measures taken were either 0.4 or 0.5 cm for this value in the unharvested tendons. Thus, this variable was essentially dichotomous, and reliability should therefore be assessed with, say, percent agreement rather than intra-class correlation.

ICC values for tendon length measurement displayed good reliability values when harvested and unharvested tendons were analyzed together (ICC _2,1_: 0.63, 4.5 ± 0.5 cm), and excellent reliability values when analyzed separately (ICC _2,1_: 0.79, 4.5 ± 0.6 cm harvested, ICC _2,1_: 0.94, 4.9 ± 0.7 cm unharvested). Hernandez et al. [26] observed greater values of patellar tendon length in professional basketball players (5.7 ± 0.6 cm), however they used the distance between patellar apex and the most prominent part of the tibial tuberosity to calculate the length of the patellar tendon measurement, instead of the notch of the tibia at the distal end as adopted in the current study.

Measurements for the tendon volume at different levels when the same examiner acquired and analyzed harvested and unharvested patellar tendons separately on separate days displayed good intra-rater reliability (ICC 2,1: 0.69 harvested, 0.67 unharvested). However, the inter-rater reliability for the same measurement ranged from poor to good over the different conditions. These findings can be partially explained by the methodology in acquiring this variable. The UTC algorithm calculates the volume based on the area of the contour that was manually drawn around the tendon by the examiner. How far within the tendon circumference one examiner decides to draw the contours affects the number of pixels within this area, thus the volume. However, a larger or smaller tendon circumference, does not affect the distribution of these pixels within the selected area.

It is important to highlight the small variability in the measurements of mid tendon thickness (approximately 0.1–0.2 cm), and in the measurements of tendon volume displayed in unharvested tendons (approximately 0.2 cm^3^). We also note that mid tendon thickness and tendon volume results should be interpreted in light of the objectively small values of the observed SEM and MDC and are approximately 10% of the grand mean. [28, 35] These parameters allow better characterization of change over time after any intervention, and given these results we recommend maintaining the same examiner for different measurements to minimize such errors.

Even though previous studies using UTC imaging have utilized different settings for analysis to quantify the proportion of echo-types of patellar tendons (window value of 25), the values of MDC observed for unharvested tendons are similar to previously published values even though the current study intentionally adopted a narrower window (17) for analysis allowing more detailed tendon tissue information. A recent reliability study in symptomatic and asymptomatic patellar tendons displayed MDC of 10.6% for echo-type I, 8.8% for echo-type II, 3.7% for echo-type III, and 2.1% for echo-type IV [10], against the 14.1% for echo-type I, 10.6% for echo-type II, 6.3% for echo-type III and 1.2% for echo-type IV calculated in this study for unharvested tendons. Interestingly, similar values of MDC were observed for harvested tendons 7.5% for echo-type I, 6.9% for echo-type II, 4.8% for echo-type III and 2% for echo-type IV.

Based on these results, future longitudinal studies could be implemented to explore possible associations of the characteristics of the patellar tendon with clinical symptoms at different time points following ACL surgery.

## Conclusions

The minimum detectable change data reported here provides some normative population specific values to allow ultrasound tissue characterization to be employed to quantify the quality of patellar tendons following ACLR. This data can then better inform any longitudinal or comparative analyses.

## Key points

### Findings

UTC imaging is a reliable tool to characterize the quality of harvested patellar tendons after ACLR and unharvested patellar tendons in patients following ACL injury.

This study provides a comprehensive description of the UTC methodology to assess and compare the quality of harvested and unharvested patellar tendons after ACL injury and/or ACLR.

### Implications

UTC imaging can be used in longitudinal studies to explore the progression of the patellar tendon tissue’s quality throughout the rehabilitation process after ACLR. Additionally, UTC imaging might be used in the future to explore possible associations of the tendon healing process with clinical symptoms at different time points following surgery.

### Caution

This is the first study using UTC to assess harvested patellar tendons after ACLR, and some aspects of the methodology used to assess the quality of these tendons differ from the methodology used for unharvested tendons. For instance, the tibial notch is not always centralized due to the harvested bone plug removed from the tibial tuberosity, and data of the distal portion of the tendon was included. Moreover, window size 17 was chosen for analysis for more detailed information of these harvested tendons rather than window size 25 as is more frequently reported. Thus, when comparing data of different studies, this difference in analysis setting should be considered.

For assessing the measurement of tendon volume over time, it is advisable that a single examiner follows the same patient throughout the period of interest.

Additional validation studies in humans are likely required to verify the echo-types classified by UTC have the same validity as those documented in horses. Finally, it should be noted that despite the growing popularity of imaging modalities in clinical practice, the relative cost of UTC imaging might be a limitation to its widespread clinical adoption.

## Data Availability

The datasets used and analyzed during the current study are available from the correspondent author on reasonable request.

## Abbreviations

ACL: Anterior cruciate ligament
ACLR: Anterior cruciate ligament reconstruction
BTB: Bone tendon bone graft
Hst: Hamstring graft
ICC: Intra-class correlation
M: Months post-operative
MDC: Minimal detectable change
Post-op: Post-operative
SD: Standard deviation
SEM % GrM: Standard error of measurement as percentage of the grand mean
SEM: Standard error of measurement
US: Ultrasound
UTC: Ultrasound tissue characterization
W: Weeks post-operative
Y: Years post-operative

## References

[1] van Schie H, Bakker E, Jonker A, van Weeren P (2000). Ultrasonographic tissue characterization of equine superficial digital flexor tendons by means of gray level statistics. Am J Vet Res.

[2] van Schie H, Bakker E, Jonker A, van Weeren P (2003). Computerized ultrasonographic tissue characterization of equine superficial digital flexor tendons by means of stability quantification of echo patterns in contiguous transverse ultrasonographic images. Am J Vet Res.

[3] van Schie H, de Vos R, de Jonge S, Bakker E, Heijboer M, Verhaar J (2010). Ultrasonographic tissue characterisation of human Achilles tendons: quantification of tendon structure through a novel non-invasive approach. Br J Sports Med.

[4] de Vos R, Weir A, Tol J, Verhaar J, Weinans H, van Schie H (2011). No effects of PRP on ultrasonographic tendon structure and neovascularisation in chronic midportion Achilles tendinopathy. Br J Sports Med.

[5] de Vos R, Heijboer M, Weinans H, Verhaar J, van Schie H (2012). Tendon Structure’s lack of relation to clinical outcome after eccentric exercises in chronic Midportion Achilles tendinopathy. J Sport Rehabil.

[6] van Schie H, Docking S, Daffy J, Praet S, Rosengarten S, Cook J (2013). Ultrasound tissue characterisation, an innovative technique for injury-prevention and monitoring of tendinopathy. Br J Sports Med.

[7] Docking SI, Rosengarten SD, Daffy J, Cook J (2015). Structural integrity is decreased in both Achilles tendons in people with unilateral Achilles tendinopathy. J Sci Med Sport.

[8] Rudavsky A, Cook JL, Docking S (2018). Proximal patellar tendon pathology can develop during adolescence in young ballet dancers- a 2 year longitudinal study. Scand J Med Sci Sports.

[9] van Schie H, Bakker E, Cherdchutham W, Jonker A, van de Lest C, van Weeren P (2009). Monitoring of the repair process of surgically created lesions in equine superficial digital flexor tendons by use of computerized ultrasonography. Am J Vet Res.

[10] van Ark M, Maciel Rabello L, Hoevenaars D, Meijerink J, van Gelderen N, Zwerver J, et al. Inter- and intra-rater reliability of ultrasound tissue characterization ( <scp>UTC</scp> ) in patellar tendons. Scand J Med Sci Sports. 2019;sms.13439. 10.1111/sms.13439. 10.1111/sms.13439 31033002

[11] Docking S, Rosengarten S, Daffy J, Cook J (2014). Treat the donut, not the hole: the pathological Achilles and patellar tendon has sufficient amounts normal tendon structure. J Sci Med Sport.

[12] Drew BT, Littlewood C, Sturrock B, Drew BT, Smith TO (2014). Do structural changes (eg, collagen/matrix) explain the response to therapeutic exercises in tendinopathy: a systematic review. Br J Sports Med.

[13] Cook JL, Rio E, Purdam CR, Docking SI (2016). Revisiting the continuum model of tendon pathology : what is its merit in clinical practice and research?. Br J Sports Med.

[14] de Jonge S, Tol JL, Weir A (2015). The tendon structure returns to asymptomatic values in nonoperatively treated Achilles tendinopathy but is not associated with symptoms. Am J Sports Med.

[15] Docking SI, van Schie J, Daffy J, Rosengarten S, Cook JL (2013). Bilateral changes in unilateral achilles tendinopathy quantified using ultrasound tissue characterisation. Br J Sports Med.

[16] Wezenbeek E, Mahieu N, Willems T, Van Tiggelen D, De Muynck M, De Clercq D (2017). What does normal tendon structure look like? New insights into tissue characterization in the Achilles tendon. Scand J Med Sci Sports.

[17] Docking SI, Ooi CC, Connell D (2015). Tendinopathy: is imaging telling us the entire story?. J Orthop Sports Phys Ther.

[18] Docking SI, Cook J (2016). Pathological tendons maintain sufficient aligned fibrillar structure on ultrasound tissue characterization (UTC). Scand J Med Sci Sports.

[19] Wong AMY, Docking SI, Cook JL, Gaida JE (2015). Does type 1 diabetes mellitus affect Achilles tendon response to a 10 km run? A case control study. BMC Musculoskelet Disord.

[20] Vaishya R, Agarwal AK, Ingole S, Vijay V (2015). Current trends in anterior cruciate ligament reconstruction: a review. Curēus.

[21] Shelbourne KD, Stube KC (1997). Anterior cruciate ligament (ACL)-deficient knee with degenerative arthrosis: treatment with an isolated autogenous patellar tendon ACL reconstruction. Knee Surg Sports Traumatol Arthrosc.

[22] Yunes M, Richmond JC, Engels EA, Pincweski LA (2001). Patellar versus hamstring tendons in anterior cruciate ligament reconstruction: a meta-analysis. Arthroscopy.

[23] Lautamies R, Harilainen A, Kettunen J, Sandelin J, Kujala UM (2008). Isokinetic quadriceps and hamstring muscle strength and knee function 5 years after anterior cruciate ligament reconstruction: comparison between bone-patellar tendon-bone and hamstring tendon autografts. Knee Surg Sports Traumatol Arthrosc.

[24] Mastrokalos DS, Springer J, Siebold R, Paessler HH (2005). Donor site morbidity and return to the preinjury activity level after anterior cruciate ligament reconstruction using ipsilateral and contralateral patellar tendon autograft: a retrospective , nonrandomized study. Am J Sports Med.

[25] Marumoto J, Mitsunaga M, Richardson A, Medoff R, Mayfield G (1996). Late patellar tendon ruptures after removal of the central third for anterior cruciate ligament reconstruction of two cases. Am J Sports Med.

[26] Hernández G, Domínguez D, Moreno J, Til L, Capdevila L (2017). Patellar tendon analysis by ultrasound tissue characterization; comparison between professional and amateur basketball players. Asymptomatic versus symptomatic. Apunt Med l’Esport.

[27] Hays WL (1988). Statistics.

[28] Denegar CR, Ball DW (1993). Assessing reliability and precision of measurement: an introduction to lntraclass correlation and standard error of measurement lntraclass correlation and standard error of measurement. J Sport Rehabil.

[29] Beckerman H, Roebroeck ME, Lankhorst GJ, Becher JG, Bezemer PD, Verbeek ALM (2001). Smallest real difference, a link between reproducibility and responsiveness. Qual Life Res.

[30] Rosengarten SD, Cook JL, Bryant AL, Cordy JT, Daffy J, Docking SI (2015). Australian football players’ Achilles tendons respond to game loads within 2 days: an ultrasound tissue characterisation (UTC) study. Br J Sports Med.

[31] Cicchetti D (1994). Guidelines, criteria, and rules of thumb for evaluating normed and standardized assessment instruments in psychology. Psychol Assess.

[32] van Ark M, Docking S, Rudavsky A, Rio E, Zwerver J, Cook JL (2016). Does the adolescent patellar tendon respond to 5 days of cumulative load during a volleyball tournament?. Scand J Med Sci Sports.

[33] Rudavsky A, Cook J, Docking S (2018). Quantifying proximal patellar tendon changes during adolescence in elite ballet dancers, a 2-year study. Scand J Med Sci Sports.

[34] Kulig K, Landel R, Chang YJ, Hannanvash N, Reischl SF, Song P (2013). Patellar tendon morphology in volleyball athletes with and without patellar tendinopathy. Scand J Med Sci Sports.

[35] Koo TK, Li MY (2016). A guideline of selecting and reporting Intraclass correlation coefficients for reliability research. J Chiropr Med.

